# Supplementary figures and images for: Pathways Related to Colon Inflammation Are Associated with Colorectal Carcinoma: A Transcriptome- and Methylome-Wide Study
Source: Cancers (Basel). 2023 May 26;15(11):2921. doi: 10.3390/cancers15112921 (PMC10251872; doi:10.3390/cancers15112921)

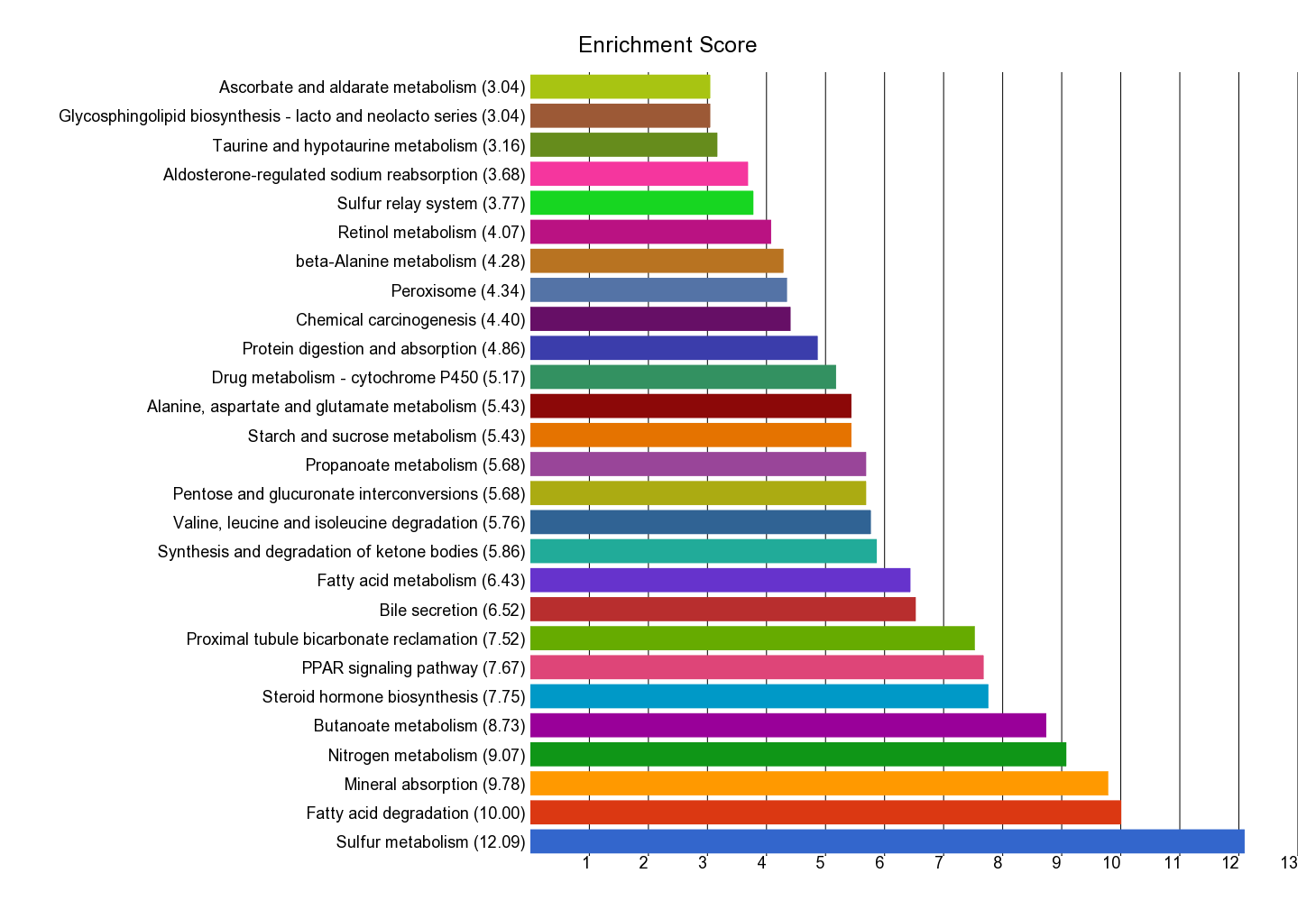

Supplement: Supplementary file 1 [file cancers-15-02921-s001.zip › Figure S1_Enrichment analysis_CPMLog2_FDR0.05_UC CRC vs Control.png]
